# Empyema Necessitatis Caused by *Prevotella melaninogenica* and *Dialister pneumosintes* Resolved with Vacuum-Assisted Closure System: A Case Report

**DOI:** 10.3390/microorganisms12091881

**Published:** 2024-09-12

**Authors:** Esteban Bladimir Martínez Castrejón, Erika Reina-Bautista, Sandra Tania Ventura-Gómez, Araceli Maldonado Cisneros, Jessica Alejandra Juárez Ramos, Miguel Alejandro Sánchez Durán, Jesús Aguilar Ventura, Omar Esteban Valencia-Ledezma, María Guadalupe Frías-De-León, Eduardo García Salazar, Carlos Alberto Castro-Fuentes

**Affiliations:** 1 Pediatric Intensive Care Unit, Hospital Regional de Alta Especialidad de Ixtapaluca, IMSS-BIENESTAR. Calle Gustavo E. Campa 54, Col. Guadalupe Inn, Alcaldía Álvaro Obregón, Mexico City 01020, Mexico; estebanmartinezcastrejon05@gmail.com (E.B.M.C.); aisely_girl@hotmail.com (A.M.C.); alehu.ice10@gmail.com (J.A.J.R.); dr_miguelsanchez@hotmail.com (M.A.S.D.); jesus.aguilar.v@hotmail.com (J.A.V.); 2 Pediatric Infectology Unit, Hospital Regional de Alta Especialidad de Ixtapaluca, IMSS-BIENESTAR. Calle Gustavo E. Campa 54, Col. Guadalupe Inn, Alcaldía Álvaro Obregón, Mexico City 01020, Mexico; erika.reinab@gmail.com; 3 Research Unit, Hospital Regional de Alta Especialidad de Ixtapaluca, IMSS-BIENESTAR. Calle Gustavo E. Campa 54, Col. Guadalupe Inn, Alcaldía Álvaro Obregón, Mexico City 01020, Mexico; esteban84valencia@gmail.com; 4 Biomedical Research Unit, Hospital Regional de Alta Especialidad de Ixtapaluca, IMSS-BIENESTAR. Calle Gustavo E. Campa 54, Col. Guadalupe Inn, Alcaldía Álvaro Obregón, Mexico City 01020, Mexico; magpefrias@gmail.com (M.G.F.-D.-L.); eduardogs01@hotmail.com (E.G.S.)

**Keywords:** empyema necessitatis, *Prevotella*, *Dialister*, VAC, case report

## Abstract

Empyema necessitatis is a rare complication of an untreated or inadequately controlled empyema. We present the case of an 11-year-old female adolescent living in precarious conditions, overcrowding, incomplete vaccinations, irregular dental hygiene, and no significant family or personal medical history. The patient started with symptoms one week prior to her hospitalization, presenting a persistent sporadic dry cough, and was later diagnosed with complicated pneumonia, resulting in the placement of an endopleural tube. Vancomycin (40 mg/kg/day) and ceftriaxone (75 mg/kg/day) were administered. However, the clinical evolution was unfavorable, with fever and respiratory distress, so a right jugular catheter was placed. The CT scan showed a loculated collection that occupied the entire right lung parenchyma and pneumothorax at the right upper lobe level. After four days of treatment, the patient still presented purulent drainage with persistent right pleural effusion syndrome. *P. melaninogenica* and *D. pneumosintes* were identified from the purulent collection on the upper right lobe, so the antimicrobial treatment was adapted to a glycopeptide, Teicoplanin, at a weight-based dosing of 6 mg/kg/day and Metronidazole at a weight-based dosing of 30 mg/kg/day. In addition, VAC therapy was used for 26 days with favorable resolution.

## 1. Introduction

Empyema necessitatis is a rare complication of untreated or inadequately controlled empyema [1]. It is characterized by the dissection of pus through the soft tissue and skin of the chest wall. The collection of pus ruptures and is released to the outside, forming a fistula between the pleural space and the skin [2,3,4,5,6,7]. The sites where empyema necessitatis most commonly extend are the anterior chest wall, esophagus, and mediastinum. Other areas described include the breast, diaphragm, retroperitoneum, and groin [7,8,9,10,11,12,13,14,15,16].

Among the main risk factors for developing empyema necessitatis are immunocompromised patients with type 2 diabetes mellitus, chronic alcoholism, chronic obstructive pulmonary disease (COPD), cachexia, poor dental health, smoking, and bronchiectasis, among others [3,6,12,17]. *Staphylococcus aureus*, *Mycobacterium tuberculosis,* and *Actinomyces* spp. are the main causative agents [2,3,4,14]. However, no cases of empyema necessitatis caused by *Prevotella melaninogenica* or *Dialister pneumosintes* have been reported in pediatric patients.

Contrasting chest tomography confirms the diagnosis, as it is highly sensitive and determines the severity and extent of the empyema [1,4,8]. Treatment of empyema necessitatis includes surgical drainage and appropriate antimicrobial treatment for infection control [1,2,7,13,14,15,16]. Another useful tool for treatment is the negative pressure applied by the VAC (Vacuum-Assisted Closure) system, which accelerates wound healing by improving blood flow in the treated area and promoting healthy growth of granulation tissue. In addition, it decreases edema and excess wound fluid and limits bacterial colonization [18].

Therefore, this work aims to present a case of empyema necessitatis caused by *P. melaninogenica* and *D. pneumosintes* in pediatric patients, which was favorably resolved with the VAC system.

## 2. Case Presentation

An 11-year-old female patient living in precarious conditions, overcrowding, incomplete vaccinations, irregular dental hygiene, and no significant family or personal medical history began experiencing symptoms one week before her hospitalization, presenting a persistent sporadic dry cough. Three days later, she presented pain in the right hemithorax, asthenia, adynamia, and dyspnea on slight exertion with no fever. On the seventh day of evolution, she presented dyspnea and cyanosis and was referred to a hospital by a physician. The patient was admitted with a diagnosis of pneumonia with complicated right pleural effusion. Two days later, a chest tube was placed, and vancomycin (40 mg/kg/day) and ceftriaxone (75 mg/kg/day) were administered. However, the clinical evolution was unfavorable, presenting fever and respiratory stress syndrome (RSS), so a right jugular catheter had to be placed. The patient was transferred to our hospital four days later with a diagnosis of possible right loculated empyema.

**Figure 1:**
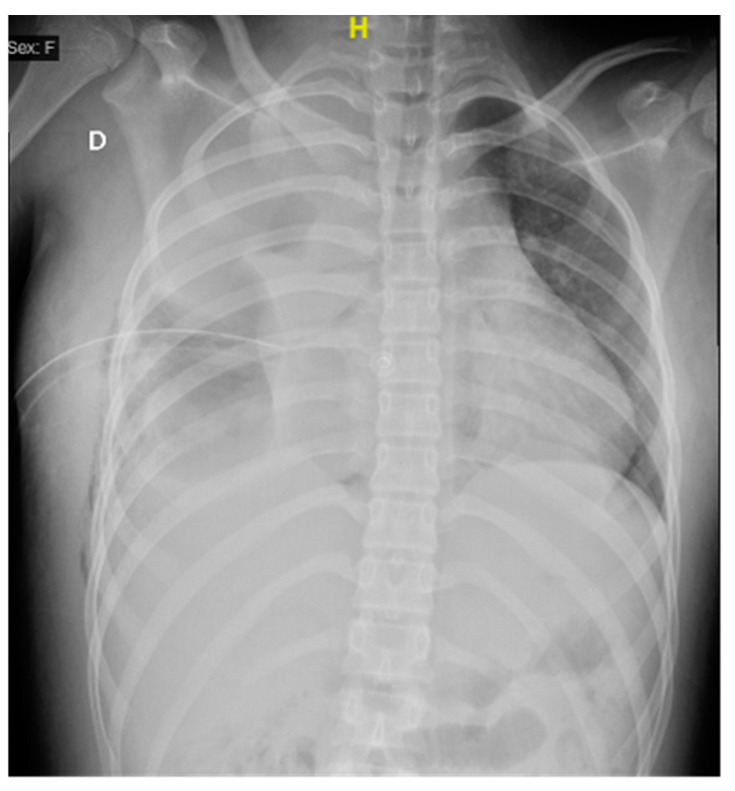
Anteroposterior thoracic X-ray taken on admission shows a right pleural tube and right pleural effusion with loss of the costophrenic angle and radiopacity in the right lung.

Upon admission to the emergency department of our hospital, she scored 15 on the Glasgow scale, requiring oxygen support with a face mask at 10 L/min, presented polypnea, the right hemithorax amplexation was decreased, and oxygen saturation of 89%. In addition, the right pleural tube showed 370 mL of hematopurulent output with a total of 128 mL (2.9 mL/kg/h) in 24 h. The gasometric study at the intake showed acid–base balance, hypoxemia, and normolactatemia (Figure 1).

**Figure 2:**
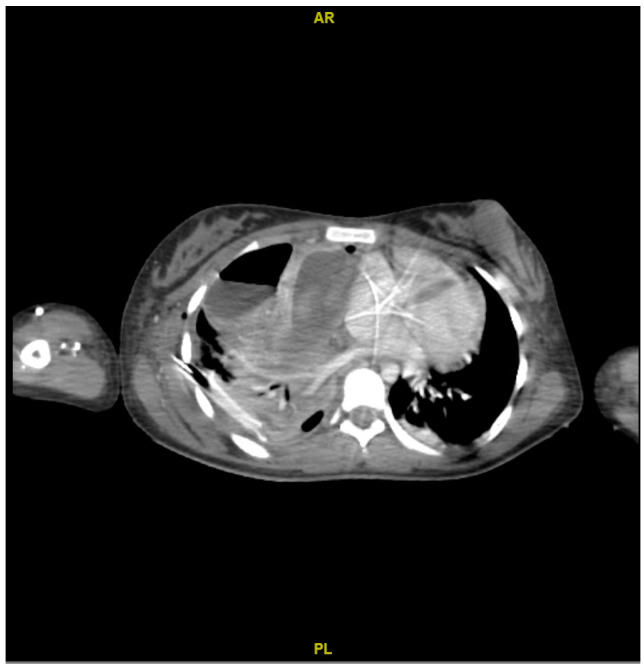
Contrasted computed tomography. Abscessified collection with hydroaeric levels, atelectasis of the parenchyma, lower zone consolidation, loculation in the base, and occlusion of the pleurostomy.

On the other hand, the computed tomography (CT) scan showed a loculated fluid collection on the entire right lung parenchyma, pneumothorax of the right upper lobe, and thickening of the right sternocleidomastoid muscle (Figure 2).

Due to the imaging findings, treatment with vancomycin and ceftriaxone was continued, covering microorganisms that cause community-acquired infectious pleural effusion. After four days of treatment, the patient continued to drain purulent output accompanied by persistent right pleural effusion syndrome. Therefore, it was decided to perform surgery that found purulent collection and fibrin on muscle and fat layers. Upon entering the thoracic cavity at the level of the fifth intercostal space, draining of apical fluid collection of aqueous characteristics occurred spontaneously, so it was considered a possible hydrothorax. A laceration area of approximately 4 cm long by 0.5 cm wide was observed on the anterior aspect of the right lower lobe. There was no active leakage at the edges, with a scarring process and fibrin on the edges, and repair was performed. A fetid purulent collection with organized clots was found in the lower lobe.

**Figure 3:**
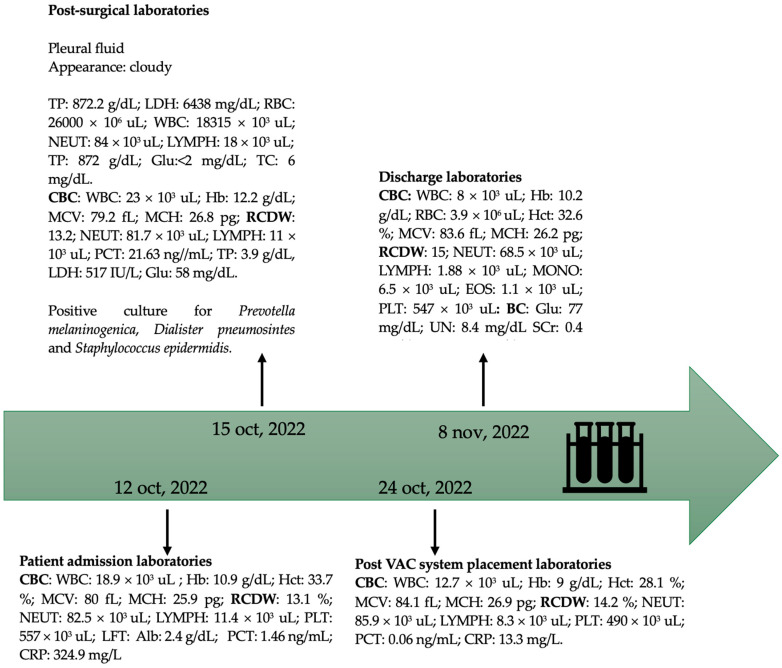
The patient’s laboratory results during the time she was treated in our hospital. Erythrocytes (RBC): 4.2–5.3 × 10^6^ uL; hemoglobin (Hb): 12.5–16 g/dL; hematocrit (Hct): 37.5–48%; leukocytes (WBC): 4.5–13.5 × 10^3^ uL; neutrophils (NEUT): 1.8–8 × 10^3^ uL; lymphocytes (LYMPH): 1.5–6.5 × 10^3^ uL; monocytes (MONO): 0–1.4 × 10^3^ uL; eosinophils (EOS): 0–0.9 × 10^3^ uL; prothrombin time (PT): 0.0 sec = 100%; C-reactive protein (CRP): 0–5 mg/L; glucose (Glu): 60–99 mg/dL; serum creatinine (SCr): 0.6–1.1 mg/dL; total cholesterol (TC): recommended less than 170 mg/dL, moderate 170–199 mg/dL, high equal to or greater than 200 mg/dL; total proteins (TPs): 6–8 g/dL; serum albumin (Alb): 3.8–5.4 g/dL; lactic dehydrogenase (LDH): 125–220 IU/L; procalcitonin (PCT): <0.1 ng/mL; medium corpuscular volume (MCV): 80–100 fL; mean corpuscular hemoglobin (MCH): 25–34 pg; red cell distribution width (RCDW): 11.5–16.6%; blood chemistry (BC): ureic nitrogen (UN): 7–16.8 mg/dL; platelets (PLTs): 130–480 × 10^3^ uL; liver function tests (LFTs).

A cytochemical study was requested, which reported cloudy purulent brown collection, glucose less than 2 mg/dL, proteins 872 g/dL, DHL 6438 IU/L, cholesterol 6 mg/dL, erythrocytes 26,000/μL, leukocytes 18,315 cell/μL, neutrophils 84%, and lymphocytes 16%, with positive light criteria for exudate (Figure 3).

Aerobic and anaerobic cultures from the collection obtained were requested, from which *Staphylococcus epidermidis* was identified in the upper collection (hydrothorax), as well as *Prevotella melaninogenica* and *Dialister pneumosintes* at anaerobic cultures. Therefore, antimicrobial treatment was adapted to a glycopeptide: Teicoplanin at a weight-based dosing of 6 mg/kg/day and Metronidazole at a weight-based dosing of 30 mg/kg/day.

**Figure 4:**
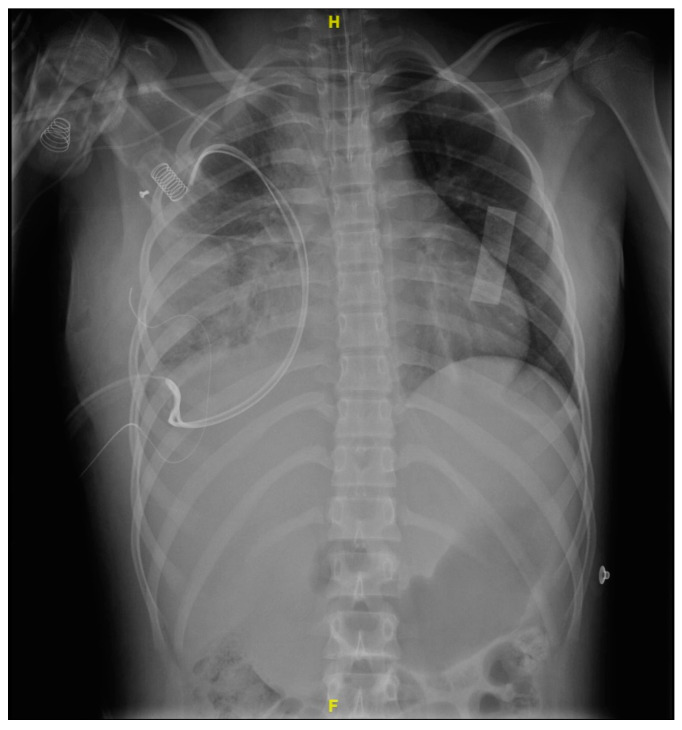
Chest X-ray after surgery (72 h after), with the persistence of multiple alveolar consolidation zones, pleural drainage, and skin drainage.

In the first 48 h after surgery, the patient continued to show data consistent with a systemic inflammatory response, with a vasopressor requirement and advanced airway management. Subsequently, due to the adequate evolution, a scheduled extubation was performed, and the patient was discharged from the intensive care unit five days after the procedure. On the following day, the thoracotomy surgical wound oozed purulent material, and wound dehiscence was noted around the endopleural tube (Figure 4).

**Figure 5:**
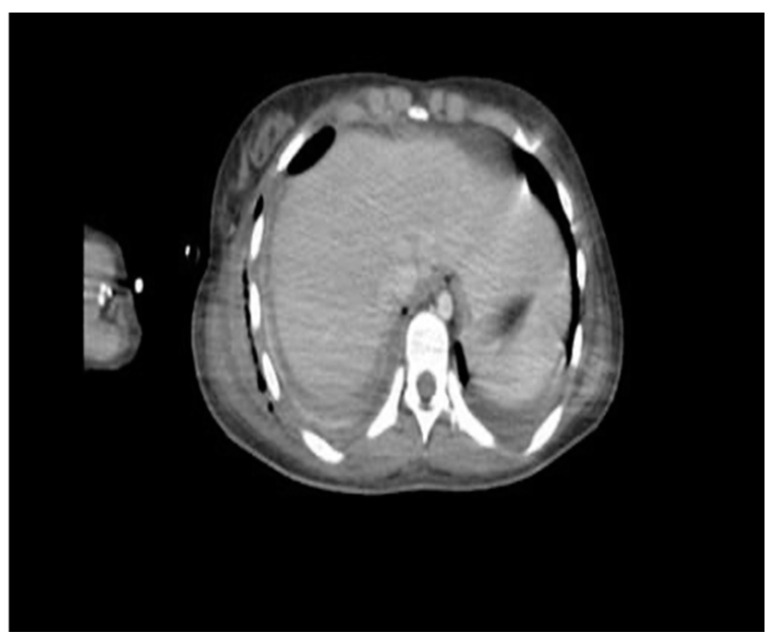
Computed tomography (CT) scan shows subcutaneous emphysema due to gas in soft tissues on the rib cage, with thickening and abscessified collection.

Therefore, the cardiothoracic surgery service re-evaluated the patient. Surgical lavage was performed to remove the pleural drainage, and a white sponge and a silver sponge with a seal were placed. The VAC system was used, opting for conservative management due to the high risk of developing a bronchopleural fistula (Figure 5).

**Figure 6:**
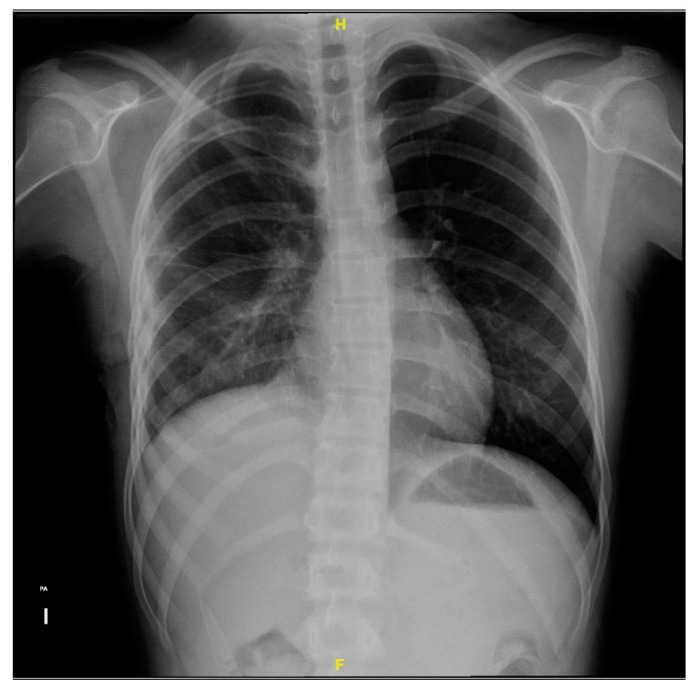
A chest X-ray taken after completely stopping the negative pressure therapy showed resolution of the infectious process, observing only persistent linear atelectasis.

VAC therapy dressings were changed every 96 h. After four replacements, rehabilitation, and nutrition management, the patient was successfully discharged home with outpatient appointments, completing management with VAC therapy for 26 days with complete remission of the pulmonary and muscular infection process (Figure 6).

## 3. Discussion

In the present case report, we highlight a case of empyema necessitatis caused by *P. melaninogenica* and *D. pneumosintes*, which was resolved by implementing the VAC system.

The incidence of empyema necessitatis is low due to the use of antimicrobials [18]. However, some cases have been reported [1,2,3,4,5,6,7]. The most common sites are the anterior chest wall, esophagus, mediastinum, and alternative extension areas, including the breast, diaphragm, retroperitoneum, and groin [2].

Approximately 40% of pneumonia in children present transudates, which can cover 10% of the pleura. Of these, 1–2% can be complicated due to exudative effusion, causing empyema [1,4]. Empyema necessitatis is more frequent in adults and immunosuppressed patients. It is a relatively rare entity in the pediatric population, with children under five years of age being the most susceptible, as with most infections [2,4].

In most of the described cases of empyema necessitatis, the main reported agents are *Mycobacterium tuberculosis* [4] or *Actinomyces* spp. [13]. Other reports include microorganisms such as *Streptococcus pneumoniae* and *Staphylococcus aureus* [2,3]. In this case, the isolated organisms were *S. epidermidis*, *P. denticola*, and *D. pneumosintes*. However, *S. epidermidis* was considered a contaminant as it is a skin colonizer. On the other hand, *P. denticola* and *D. neumosintes* were not considered contaminants as they are colonizers of the oral cavity; besides, they can promote aspiration pneumonia [9]. Therefore, *P. denticola* and *D. pneumosintes* were determined to be our patient’s causative agents of empyema.

Most *Prevotella* sp. organisms are recovered from the oral cavity of humans, with *P. melaninogenica* being the main species of the genus. In healthy adults, detection rates for *Prevotella* spp. are high, particularly in saliva and dental plaque. In the respiratory tract, potential routes for bacterial translocation from the oral cavity include microaspiration and hematogenous dissemination. Currently, the genus *Prevotella* is considered one of the primary colonizers of the mucosal surfaces of the aerodigestive tract [5]. Although oral bacteria access the proximal airways by microaspiration, continuous mucociliary clearance prevents their growth in high densities. Oral bacteria present in saliva can promote aspiration pneumonia by colonizing mucosal surfaces, thereby affecting the immune response of epithelial cells and producing pro-inflammatory cytokines and degrading enzymes [9].

On the other hand, *D. pneumosintes* is a small, non-fermentative, rod-shaped Gram-negative bacillus. Currently, there are four known species of the genus *Dialister*, consisting of 135 strains; however, *D. pneumosintes* and *D. microaerophilic* are commonly found species. Proof of this are reports of serious infectious complications, including severe pneumonia and sepsis, brain abscesses, and liver abscesses suspected of dental origin [19,20].

In pneumonia and pleural empyema, both anaerobes and oral bacteria can go unnoticed in conventional culture and are found more frequently by molecular methods [9]. This is the case of *D. pneumosintes*, an organism difficult to culture in conventional media but identifiable through the 16s rRNA-based PCR (polymerase chain reaction) assay, which suggests anaerobic Gram-negative bacilli [20]. In the present case report, the isolation of *D. pneumosintes* was performed in a culture medium for anaerobes. In this sense, it is recommended to consider a bacterial growth time of 34 h to determine the diagnosis and treatment for the patient [19]. However, through the PCR assay, the identification of *D. pneumosintes* has been achieved in cases of periodontitis, gingivitis, subgingival plaque, respiratory tract infection, head and neck, and vaginal infection [20].

Our patient developed hemithorax, asthenia, adynamia, and dyspnea. A few days later, she developed a fever and respiratory stress syndrome. This corresponds with the report by Kaiser et al. [19], who described the case of a 13-year-old patient admitted for sepsis of unknown origin. Like our patient, due to the worsening of her lung condition, she required intubation. Through CT, severe pneumonia and an emergency due to respiratory stress syndrome were identified in our patient. Therefore, antibiotic treatment was started.

The surgical management of empyema necessitatis is imperative. In this case, we opted for the VAC system because, among other benefits, it accelerates wound healing by improving blood flow in the treated area and promoting the healthy growth of granulation tissue [18]. In addition, it reduces edema and excess wound fluid and limits bacterial colonization [21], allowing our patient’s favorable evolution. In addition, intrathoracic application of the VAC system may result in a shorter period of hospitalization [22], as this treatment can be provided on an outpatient basis. So far, reports have shown that patients can completely recover after using the VAC system in the chest [18,21,22,23].

According to the review of the literature carried out in the main databases such as PubMed, Scopus, and Scielo, to date, there is no information on the resolution of empyema necessitatis caused by *Prevotella melaninogenica* and *Dialister pneumosintes* resolved with the VAC system in a pediatric patient. This is, to our knowledge, the first case report on this topic.

## 4. Conclusions

Our patient’s empyema necessitatis may have had an oral origin due to poor hygiene, being caused by the precarious living conditions of developing countries.

The identification of *P. melaninogenica* and *D. pneumosintes* was performed by anaerobic cultures and not by PCR. Therefore, it represents a highly useful diagnostic tool.

It is essential to obtain a detailed medical history and assess individually the risk factors predisposing each patient to this disease. Furthermore, *D. pneumosintes* and *P. melaninogenica* should be considered as causative agents of empyema necessitatis.

The implementation of the VAC system allowed our patient to progress favorably. Therefore, this system represents an alternative for treating empyema necessitatis in pediatric patients.

## Data Availability

Data are contained within the article.
